# Gold, Gold-Beating, Compounds and Alloys

**Published:** 1853-07

**Authors:** R. N. Wright


### ARTICLE III.

### *Gold, Gold-beating, Compounds and Alloys.* By Professor R. N. Wright, M. D.

(Continued from page 400.)

### SILVER.

This metal, (the second of our proposed series,) holds a place in the catalogue of "old metals." It has been known from remote antiquity, as is proven by the fact of its frequent mention by the earliest writers, both sacred and profane. The ancients, whose list of metals was by no means extensive, named them from their physical appearances, after the planets of our solar system, in accordance with which, from its peculiarly soft lustre, silver was called "luna," hence the common use at the present day of the term "lunar," when referring to the preparations of silver.

The present supply of silver, as compared with gold, is limited—it is, however, found in various parts of the world in considerable quantity, both in the form of "native silver" and in combination, occuring most commonly in the latter form, lead being the metal with which it is found most frequently combined.

The mines of South America and Mexico are the richest in the world—those of Northern and Central Europe, the riehest in the old world, among which those of Saxony, stand perhaps, preeminent.

Native silver, if scratched or rubbed, presents the usual characteristics of silver, being very bright and susceptible of a high polish. The form of the mass is quite peculiar, sometimes exhibiting numerous crystalline facets, but more commonly exhibiting a rough thready appearance. I have seen specimens of native silver, from Mexico, which presented such an appearance as would have been manifested, had they been melted, and while fluid, rapidly poured into cold water, thus giving them a very loose and fibrous texture.

*Methods of reducing the Ores of Silver.*—Of the various processes for the reduction of silver ores, I will mention but three, viz. *eliguation*, *amalgamation* and *cupellation*. The first of these, *eliguation*, is now among the old and disused processes. It consists in making a triple alloy of copper, lead and silver. It is made into masses of a convenient form and size, these to be subjected to the action of heat in furnaces of peculiar construction, the bottom being of iron and fluted and grooved, and inclined in such a manner as to allow any melted metal to run readily off. Lead and silver have a strong affinity for each other, and consequently when the former melts the latter is carried along with it. The copper, in an altered condition remaining in the furnace.

*Amalgamation*, according to the best authorities, is accomplished as follows: the ore to be subjected to the process, is first washed, then ground, it is now roasted, after having been mixed with *chloride of sodium;* by this treatment we have in the resuling mass, two new compounds, viz. *chloride of silver* and *sulphate of soda.* Three other substances are next added, viz. *water*, *iron*, *(in fine division)* and *mercury*—and the whole mass after having been pulverized, is agitated in barrels made to revolve rapidly; one of the substances formed by this process is chloride of iron, which is washed out; the silver now combines with the *mercury* forming an *amalgam*—which is next separated by the action of *heat*, the *mercury* being thus evaporated; and in order to prevent loss, it is best to conduct the process in an *iron retort* with its beak opening into a condenser; here the mercury may be received, and reserved for future use. It sometimes happens, that there is an excess of *mercury*, this is got rid of by placing the *amalgam*, (before distillation,) in leather bags, and subjecting it to pressure, by which the excess is removed.

*Cupellation* consists in subjecting the alloy containing the silver to the action of heat in a current of air, the mass being placed in a porous crucible or "cupel," (as it is called,) which absorbs the remaining lead and leaves a button of pure silver. We will, however, describe this process more fully presently.

Mr. Graham, page 458, gives the following methods for obtaining silver in a pure state for "chemical and other purposes:" 1. "The metal is dissolved in pure *nitric acid, diluted slightly*, and precipitated by a solution of *chloride of sodium;* the salts of the other metals present, remain in solution. The insoluble *chloride of silver* thus obtained, is washed well upon a filter with hot water, and dried. A quantity of *carbonate of potash*, equal to *twice* the weight of the *silver*, is fused in a crucible, and the *chloride of silver* gradually added to it; *chloride of potassium* is formed, and *carbonic acid* and *oxygen gas* are given off, with effervescence. The crucible is then exposed to a sufficient heat, to fuse the reduced silver, which subsides to the bottom." 2. "The mode of separating *silver* from the common metals, in the ordinary practice of assaying, is like many metallurgic operations, an exceedingly elegant and refined process. A portion of the *silver alloy*, the *assay*, is *fused*, with several times its weight of *pure lead*, (an alloy of 1 copper and 15 silver, with 96 lead for instance,) upon a *bone-earth cupel*,* which is supported in a little oven or muffle, heated by a proper furnace. Air being allowed access to the *assay*, the *lead* is rapidly *oxydated*, and its highly fusible oxyd imbibed, as it is produced by the porous cupel. The disposition of *copper*, and other common metals to *oxydate*, is increased by the presence of the *lead*, and their oxyds, which form fusible compounds with oxyd of *lead*, are removed in company with the latter. When the *foreign metal* is nearly entirely removed, the *assay* is observed to become rounder and more brilliant, and the last trace of fused oxyd, occasions a beautiful play of prismatic colors upon its surface, after which, the assay becomes, in an instant, much whiter, or *flashes*, an indication that the cupellation is completed."

The *specific gravity* of silver, will be found varying, increasing or diminishing according to the process to which it has been subjected. When cast and not subjected to pressure, it will be found to have a density of about 10-4—which may be increased by the rolling mill or hammer to 10-5 and even to 10-6. Its melting point is commonly stated at 1873°, a bright red heat, its ductility is such, that it can be drawn into wires as fine as

* Already referred to in a former paragraph.

a very light silk thread, and by careful hammering, it can be beaten into leaves, not exceeding the 10,000th of an inch in thickness; it is a good conductor of heat and electricity, an electrical discharge passing along it silently, if the mass of metal have sufficient thickness, but if in the shape of silver leaf, it undergoes combustion, changing into a dark colored powder; combustion effected by means of a galvanic current, is attended with the appearance of bright green light. Silver, while fluid, seems to possess a strong power of absorbing oxygen gas, which it gives off when the metal is cooled rapidly, giving rise to the production of a phenomenon, which assayists call "spitting," and it does not appear in this instance that the oxygen has combined chemically with the metal, but that it has been simply absorbed.

In speaking of this matter, Brande says, "this property of absorbing oxygen is prevented by the presence of a quantity of copper, not exceeding five per cent. When nitre is thrown upon melted silver, in a crucible, and the whole retained for about half an hour in fusion, it absorbs twenty times its volume of oxygen, which is given out, on plunging the fused metal under a bell jar filled with water." Silver is not acted on by the oxygen of the air under ordinary circumstances, though it is readily discolored, when sulphureted hydrogen is present.

Graham has, upon this subject, the following paragraph, page 459:

"Silver does not combine with the oxygen of the air at the usual temperature, not even when heated; the tarnishing of polished silver in air, is occasioned by the formation of sulphuret of silver. Silver does not dissolve in any hydrated acid, by substitution for hydrogen, but on the contrary, it is displaced from a solution in an acid by hydrogen, and precipitated in a metallic state. This metal is also precipitated by mercury, and all the more oxydable metals. Its salts are reduced at the usual temperature, by sulphate of iron, of which, the protoxyd is converted into the peroxyd. But if the persulphate of iron be boiled upon the precipitated silver, the latter is dissolved again, and oxyd of silver and protoxyd of iron reproduced.

Silver, however, is oxydated when fused or heated strongly in contact with substances for which oxyd of silver has a great affinity; as with a siliceous glass, and stains the glass yellow. It is oxydated by boiling concentrated sulphuric acid with the escape of sulphurous acid. Silver is readily dissolved by nitric acid, with a gentle heat, and with much violence; at a high temperature, nitrate of silver is formed and nitric oxyd escapes."

We will next proceed to detail some of the more important combinations which silver enters into with other substances—and first of all with other metals, or the *alloys of silver*. For this, together with the table of the weight, value, &c. of British silver coins, we are indebted to Brande, and inasmuch as we can scarcely alter his paragraph so as to improve it, we will allow him to speak for himself, by printing his account verbatim; he says, "the compounds of silver with *potassium*, *sodium* and other light metals have not been examined, neither has it been alloyed with manganese. It unites difficultly with iron. Upon fusing silver with iron, the alloy separates into silver, retaining about one-thirtieth of iron, and iron retaining about one-eightieth of silver; the latter has a peculiar, hard and crystalline texture. (Morveau. Journ. de Phys., 1788.) When silver and steel are fused together, an alloy is formed which appears perfect while in fusion, but globules of silver exude from it on cooling, which shows the weak attraction of the metals. At a very high temperature, the greater part of the silver evaporates, but a portion equal to about 1 in 500 remains, forming a perfect alloy, admirably adapted to the formation of cutting instruments. (Stodart and Faraday, Quarterly Journal, ix.) Silver readily combines with zinc, producing a brittle bluish-white, granular alloy. When an alloy of 11 parts of zinc, and 1 of silver, is highly heated in an open crucible, it burns, and the whole of the silver is sublimed with the oxyd of zinc. With *tin*, silver forms a white, hard brittle alloy. Silver made standard by tin, is brittle, and does not ring well. (Hatchett, Phil. Trans., 1803.) The alloy with *cadmium*, has not been described. When *cobalt* and silver are fused together, the metals separate on cooling, each retaining a small portion of the other; the silver is rendered brittle, and the *cobalt* white. (Gellert.) The alloy of silver and *nickel*, has not been examined. The alloy with *copper*, constitutes plate and coin:* by

* *Table of the Weight, Value, &c. of British Silver Coin.*

| Numbers of pieces in the ℔ troy. | | Standard weig't of each piece. | | Fine silver in each piece. | | Allowance for current wear. | Remedy by indenture, August 16, 1817, above or below standard. | | Working remedy for the Monayers, by mint regulation. | | | | | Legal Tender | Standard weight in troy grains. |
|---|---|---|---|---|---|---|---|---|---|---|---|---|---|---|---|
| | | | | | | | Weight | Fineness. | On each piece above or below standard. | Heaviest piece. | | Lightest piece. | | | |
| | | dwt. | gr. | dwt. | gr. | | dwt. | dwt. | gr. | dwt. | gr. | dwt. | gr. | | gr. |
| 792 | Pence. | | 7.27 | | 6.72 | Allowed to pass current without any limitation as to weight. | 1. | 1. | .03 | | 7.30 | | 7.24 | | 7.272 |
| 396 | Twopences. | | 14.54 | | 13.45 | | | | .06 | | 14.60 | | 14.48 | | 14.545 |
| 264 | Threepences | | 21.81 | | 20.18 | | | | .09 | | 21.90 | | 21.72 | | 21.818 |
| 198 | Fourpences. | 1. | 5.09 | 1. | 2.90 | | | | .12 | 1. | 5.21 | 1. | 4.97 | | 29.090 |
| 132 | Sixpences. | 1. | 19.63 | 1. | 16.36 | | | | .18 | 1. | 19.81 | 1. | 19.45 | 40s. | 43.636 |
| 66 | Shillings. | 3. | 15.27 | 3. | 8.72 | | | | .36 | 3. | 15.63 | 3. | 14.90 | | 87.272 |
| 26 & 1sh | ½ Crowns. | 9. | 2.18 | 8. | 9.81 | | | | .90 | 9. | 3.09 | 9. | 1.27 | | 218.181 |
| 13 & 1sh | Crowns. | 18. | 4.36 | 16. | 19.63 | | | | 1.81 | 18. | 5.18 | 18. | 2.54 | | 436.363 |

The assays of silver are reported in ounces and pennyweights.

The lowest, is ½ dwt. Deliveries of coin are made by the Monayers to the mint office, in journey weights; each journey, 60 lbs. troy, in value £198.

the addition of a small proportion of copper to silver, the metal is rendered harder and more sonorous, while its color, is scarcely impaired. Even with equal weights of the two metals, the compound is white ; the maximum of hardness is obtained, when the copper amounts to one-fifth of the silver. The standard silver of this country (England) consists of $11\frac{2}{20}$ pure silver, and $\frac{18}{20}$ copper, or 11-10 silver and 0-90 copper. A pound Troy, therefore, is composed of 11 oz. 2 dwts. pure silver, and 18 dwts of copper. Its density is 10-3 ; its calculated density is 10-5, so that the metals dilate a little on combining. The French silver coin is constituted of 9 silver and 1 copper ; the silver coins of the ancients, and many oriental silver coins are nearly pure, they only contain traces of copper and gold. When silver alloyed by copper, such as standard silver, is exposed to a red heat in the air, it becomes black, from the formation of a superficial film of oxyd of copper ; this may be removed by immersion in hot diluted sulphuric acid, and a film of pure silver then remains, of a beautiful whiteness ; this is called *blanched* or *dead* silver ; the blanks for coin are treated in this way before they are struck, whence the whiteness of new coin, and the darker appearance of the projecting portions occasioned by wear, in consequence of the alloy showing itself beneath the pure surface ; articles of plate are often *deadened* or *matted* by boiling in bi-sulphate of potassa, (sal enixum,) which acts in the same way as the dilute sulphuric acid.

French standard silver, is an alloy of 9 of silver and 1 of copper. *Lead* and silver form a very brittle dull colored alloy, from which the lead is easily separated by *cupellation.* When fused lead, containing silver, is suffered to cool slowly, the lead, which first concretes, forms granulary crystals and is nearly pure, while almost the whole of the silver is contained in the liquid portion ; in this way the separation of the two metals may to a certain extent, be effected, especially upon the large scale. *Antimony* forms a brittle white alloy, the density of which, exceeds the mean of its components, the greater part of the antimony evaporates during protracted fusion, and the whole may be separated in the form of oxyd by roasting. There is a rare ore found in Spain, and Suabia, (*antimonial silver*,) which has a white metallic lustre, and contains 77 per cent. of silver, and 23 of antimony; its density is 9·82. ***Bismuth*** and silver may be combined by fusion; the alloy is brittle, yellow-white, and lamellar; its density exceeds the mean; the density of an alloy of equal weight of bismuth and silver is 10·7. The alloys with *uranium*, *titanium*, *cerium* and *tellunium* are not known. When silver and arsenic are fused together, an alloy is formed, composed of 100 silver + 16 arsenic. (Gehlen.) It is grey, brittle, granular, and by long fusion, great part of the arsenic evaporates; it may be entirely got rid of by roasting. Arsenuret of silver is found *native.* Four parts of silver, and two of *molybdenum* were strongly heated, but did not yield a button; by continuing the heat, a portion of the silver eliquated, still retaining a part of the molybdenum, and becoming bluish when heated: the residuum being again melted in charcoal became more compact, was brittle, grey and granular. (Thomson.) The alloys of *chromium* and *vanadium*, are unknown. 100 parts of silver and 50 of *tungstic acid*, strongly heated with charcoal, gave a brown button, slightly malleable, weighing 142 grains. (Thomson.)

Silver amalgamates easily with *mercury ;* when red hot silver is thrown into heated mercury it dissolves, and when 8 parts of mercury and 1 of silver are thus combined, a granular crystalline soft amalgam is obtained, the density of which exceeds the mean of its components; when a solution of this amalgam in liquid mercury is squeezed through chamois leather, the excess of mercury, retaining only a trace of silver, goes through, and the solid amalgam is left behind. This amalgam exists native, in octoëdral and dodecaëdral crystals, containing 34-65 per cent. of mercury. Amalgam of silver is sometimes employed for plating; it is applied to the surface of copper, and the mercury being evaporated by heat, the remaining silver is burnished. The better kind of plating, however, is performed by the application of a plate of silver to the surface of the copper, which is afterwards beaten or drawn out. A mixture of chloride of silver, chalk and pearlash, is employed for silvering brass; the metal is rendered very clean, and the above mixture moistened with water rubbed upon its surface. Plating by metallic precipitation from an ammonio-chloride of silver, and also by voltaic precipitation, is now coming into use."

The latter process will be described at length in the end of the present article.

Having progressed thus far with the consideration of our subject, we propose next to commence the examination of substances formed with non-metallic elements and compounds, commencing the list with the oxyds; of which there are three, according to the best authorities.

1. *Suboxyd.* (2 Ag. + o.)—The existence of this oxyd, is now established beyond question, and for it we are chiefly indebted to the experiments of Wöhler. His process consisted in exposing citrate of silver to the action of hydrogen gas, at a high temperature, about 212° F. One-half the oxygen in the above combination becomes disengaged from the base, while the other half remains, and with this new suboxyd the citric acid combines, the forming a *citrate of the suboxyd of silver.* Mr. Faraday's process for obtaining it is different; he proposes to expose a solution of the protoxyd in ammonia, to the action of the air, when the substance in question separates. When the dark brown solution of suboxyd of silver is heated, the solution becomes colorless, and silver makes its appearance in the metallic state.

2. *Protoxyd.* (Ag. + o.)—The proper method for procuring this substance, is to pour into a solution of the *nitrate of silver* in *distilled* water, a strong solution of some *alkali* or *alkaline earth*—say *potash or lime*—immediately upon the addition of one of these substance, a dark olive colored precipitate makes its appearance, and slowly subsides to the bottom of the glass. After all deposition has ceased, the contents of the glass may be thrown on a filter, where the precipitate is to be carefully washed. Graham says, it is partially soluble in water, and under favorable circumstances, when so dissolved, possesses an alkaline reaction. The principal use to which this substance has been put in the arts, is in imparting a yellow color in enamel and porcelain painting. It should be preserved in bottles from which all light is carefully excluded, for, in course of time it undergoes partial decomposition by such exposure, being probably converted into suboxyd and metallic silver. The application of heat will effect its reduction.

3. *Peroxyd.* (Ag. + 20,) or (ag. + 02,) with regard to this substance, Brande has the following paragraph: "Ritter, by electrizing a weak solution of silver, observed the deposition of acicular crystals at the positive pole, which, according to Grotthuss, dissolve in nitric acid, without decomposition, and are a *peroxyd of silver*. Ammonia energetically decomposes this compound, and sulphuric and phosphoric acids, convert it into the *protoxyd.*" (Gehlen's Jour., iii.)

*Iodide of Silver.*—Vauquelin has discovered this substance native, in some of the specimens of ores from the Mexican mines, but in very minute quantities. The best method which can be adopted for its formation, artificially, is to call to our aid *elective affinity;* by adding to a solution of *nitrate of silver* in *distilled water*, a solution of *hydriodate of potash*, a dirty looking precipitate of a yellowish color is deposited, which undergoes change of color by exposure to light and high temperature, the latter imparting a red color. The singular and permanent effects produced upon this iodide by light, led to the discovery of Daguerre's method of making pictures—"Photography;" the plate upon which the image is to be made, after having been well cleaned, is exposed to the vapor of iodine, in consequence of which, the surface becomes covered with a thin pellicle of iodide of silver, and is ready for the camera. The plates used in the above process, are commonly but silvered copper. (ag. + i.) represents its chemical constitution.

*Bromide of silver* very closely imitates the iodide in its formation, and the method of preparation; for by adding a solution of *bromide of potassium*, to one of nitrate of silver, a yellowish precipitate makes its appearance, very much resembling the iodide, but it may easily be distinguished, by the fact that, the *bromide* is readily soluble in ammonia, whereas the *iodide*

is remarkable for its insolubility in the same—(Ag + b.) represents its chemical constitution.

*Fluoride of silver* is the next compound in regular order, it is, however, a compound of little consequence, and, perhaps, it is enough to say of it, that it is uncrystallizable, and that (ag + f.) indicates its chemical constitution.

The next compound we will present to the consideration of the reader, is one of much more importance than either of the three just described, viz. that combination of *silver and chlorine gas*, known as

*Chloride of silver*, which can best be prepared, by adding a solution of *chloride of sodium*, to one of *nitrate of silver* in distilled water. A dense precipitate is the instantaneous result, white at first, but, by exposure to light, passing through a variety of shades, from light purple to a very dark brown, and the rapidity with which this change of color takes place, depends in great measure upon the vividness of the light to which it is exposed, an effect which is somewhat retarded, if *mercury* in minute quantities be present, when the experiment is made.

In connection with this subject, it may not be amiss to introduce in this place, a few paragraphs, which will be found in Brande, (page 200,) he says, "some of the salts of silver, especially their chlorides, are remarkably susceptible tests of the chemical agency of *light;* the *nitrate of silver* may be selected by way of illustration. If a piece of paper be dipped into a solution of this salt, and kept in the dark, it suffers no apparent change; but if exposed to light, it soon becomes purple, brown and black, changes of color depending upon a chemical change, suffered by the salt. A pretty experiment, showing the action of light upon nitrate of silver, was devised by Mr. Wedgwood; a piece of paper, or other convenient material, was stretched upon a frame and sponged over, with a solution of the salt, it was then placed behind a painting upon glass, and the light traversing the painting, produced a kind of copy of it upon the prepared paper, those parts in which the rays were least intercepted, being of the darkest hues."

"Scheele was the first to whom the ingenious idea occurred, of ascertaining whether all the rays possessed similar *chemical* powers, or whether they belonged, more exclusively to one color than to another; and he found, upon refracting a beam by the prism into its primary colors, and throwing them upon a piece of paper prepared with nitrate of silver, that the greatest blackening effect was produced by the violet ray; and, that the decomposing or chemical powers of the prismatic spectrum, gradually decreased towards the *red* ray, when *scarcely any effect* was produced. This effect was quite contrary to expectation; for one would of course, have anticipated the greatest effect, in the most luminous part of the spectrum."

"It was afterwards discovered by Wollaston and Ritter, (Phil. Trans. 1802, and Philos. Journal, iv and viii,) that certain invisible rays, occupying a place in the spectrum, just beyond the *violet* extremity, possessed a greater power of effecting the above and greater chemical changes, than the violet rays themselves; and, it has, consequently, been inferred, that such distinct rays emanate from the sun, possessed, as their place in the spectrum shows, of great refrangibility; and that the colored rays (and their mixture, constituting white light,) derive their chemical powers from the admixture of these highly refrangible and chemically acting rays, which are most abundant at the blue end of the spectrum, and gradually decrease towards the red, or least refrangible rays. Thus, it was found, that the greatest blackening effect upon the *salt of silver*, was produced just beyond, and out of the violet ray; it must, however, be observed, that the place occupied by these rays in the spectrum, depends, in some measure, upon the nature of the medium by which the light is refracted."

In the above process, there seems to be a partial *reduction* of the metal, and we must infer the presence of *water*, from the fact, that some *muriatic acid* is simultaneously produced.

The presence of *sulphuric acid*, when *chloride of silver* is kept with it in tight bottles, seems to have the effect of materially retarding the change in colors of which we have just been speaking.

Chloride of silver, is nearly or quite insoluble in water, and hence it sometimes renders us valuable aid in quantitative analysis; for by forming a precipitate very carefully, we can estimate the amount of chlorine present in solution and in making this precipitate, we should allow the supernatent liquid to become perfectly clear before it is decanted. The chloride after having been thus obtained, should be dried in a silver crucible at a high temperature. To facilitate the formation of a precipitate, it may sometimes be necessary to use heat or to add a small quantity of acid; nitric being most commonly used. Ammonia dissolves chloride of silver with extreme readiness, thus furnishing us with a distinctive characteristic, between this and other chlorides of the same color. A fulminating compound is formed, sometimes on the application of heat to these solutions in ammonia. Faraday says, that if *dry chloride of silver* be saturated with *ammonia*, and thrown into chlorine it will immediately kindle into flame. A solution in some hyposulphate in the liquid form, or in chloride of potassium—or hydrochlorate of ammonia, may be used for silvering the surfaces of metals, as copper—(ag. + cl.) will represent the chemical constitution of chloride of silver.

*Cyanuret of Silver*, is a compound produced whenever hydrocyanic acid is added to a solution of nitrate of silver; a white precipitate of greater or less density falling, its density being regulated by the strength of the solutions used.

*Ferro-cyanuret of Silver*, is found wherever we add a solution of *ferro-cyanuret of potassium* to one of nitrate of silver; it is a dense white powder, and unimportant.

*Sulphur-cyanuret of Silver*, is in like manner produced, when sulpho-cyanuret of potassium is added to a solution of nitrate of silver; it is white when first produced, but gradually changes colors by exposure to light; it is unimportant.

*Sulphuret of Silver*, especially when in the condition of *native sulphuret*, is a very important substance. Artificially it may be prepared in two ways, either by mixing *sulphur* and *silver* and applying heat, or by passing a stream of *sulphureted hydrogen gas* through a solution of one of its soluble salts, as the nitrate; the precipitate so produced, is dark colored and very dense. In order to preserve it for use, the contents of the precipitation glass should be thrown on a filter and the precipitate carefully washed and dried.

The tarnishing of silver, which is so commonly noticed, especially where it is exposed to the action of the atmosphere of large cities, is occasioned by the presence of *sulphureted hydrogen gas*, which is always to be found, in greater or less quantity under such circumstances, eliminated from the many sewers and other similar places, where decomposition is going on. Some of the most beautiful native sulphurets of silver, are found in Northern Asia. Sulphuret of silver is represented chemically by (ag. + s.)

*Seleniuret of Silver*, is formed whenever a stream of seleniureted hydrogen gas is made to pass through nitrate of silver in solution, it consists of one atom of each of its constituents, but is too unimportant to need further comment.

*Phosphuret of Silver*, is thus described by Brande, "it is a brittle compound; is either formed by projecting phosphorus upon red hot silver, or by heating a mixture of 1 part of silver filings, 2 of vitrified phosphoric acid, and 0.5 of charcoal. It loses its phosphorus when fused and exposed to air. Pelletier observed, in regard to this phosphuret, that the fused silver was capable of retaining a larger proportion of phosphorus in combination than after it had solidified; for the fused phosphuret, when it concretes, throws off a quantity of phosphorus, producing a brilliant combustion; there is an analogy between this effect, and the similar absorption and evolution of oxygen. The concerted phosphuret consists 108 silver + 16 phosphorus." (Ag. + p.) represents its chemical constitution.

*Ammoniuret of Silver.*—Berthollet, who discovered and has described this substance, gives the following process for its preparation: pour a small quantity of liquid ammonia upon the oxyd, a portion is dissolved, and a detonating black powder is left; potassa will precipitate it from *ammonio-nitrate of silver*. When gently heated, (he goes on to say,) it explodes, *nitrogen* and water are evolved immediately, attended with the reduction of silver. Pure oxyd of silver, and ammonia free from carbonic acid, are required for the successful performance of the experiment. Brande says, "It should only be made in small quantities, and handled with the greatest caution, many accidents having arisen from its careless management. It sometime explodes while still wet. It is soluble in ammonia; it is immediately decomposed by hydrochloric acid, which forms chloride of silver and salammoniac, and by sulphureted hydrogen, which forms sulphuret of silver and hydro-sulphuret of ammonia; with sulphuric acid, it affords sulphate of silver and of ammonia, but nitrogen is also evolved. The real nature of this compound is, probably, not at present understood."

[To be concluded in the next No.]

### ARTICLE IV.

*On the Morbid Changes of Saliva.* By Prof. A. SNOWDEN PIGGOT, M. D.

